# Short-Term Trajectories of Use of a Caloric-Monitoring Mobile Phone App Among Patients With Type 2 Diabetes Mellitus in a Primary Care Setting

**DOI:** 10.2196/jmir.3938

**Published:** 2015-02-03

**Authors:** Glenn Goh, Ngiap Chuan Tan, Rahul Malhotra, Uma Padmanabhan, Sylvaine Barbier, John Carson Allen Jr, Truls Østbye

**Affiliations:** ^1^Duke-NUS Graduate Medical SchoolSingaporeSingapore; ^2^SingHealth PolyclinicsSingaporeSingapore; ^3^Duke Global Health InstituteDurham, NCUnited States; ^4^Health Promotion BoardSingaporeSingapore

**Keywords:** type 2 diabetes mellitus, self-management, mobile phone, mobile apps, longitudinal studies

## Abstract

**Background:**

Self-management plays an important role in maintaining good control of diabetes mellitus, and mobile phone interventions have been shown to improve such self-management. The Health Promotion Board of Singapore has created a caloric-monitoring mobile health app, the “interactive Diet and Activity Tracker” (iDAT).

**Objective:**

The objective was to identify and describe short-term (8-week) trajectories of use of the iDAT app among patients with type 2 diabetes mellitus in a primary care setting in Singapore, and identify patient characteristics associated with each trajectory.

**Methods:**

A total of 84 patients with type 2 diabetes mellitus from a public primary care clinic in Singapore who had not previously used the iDAT app were enrolled. The app was demonstrated and patients’ weekly use of the app was monitored over 8 weeks. Weekly use was defined as any record in terms of food entry or exercise workout entry in that week. Information on demographics, diet and exercise motivation, diabetes self-efficacy (Diabetes Empowerment Scale-Short Form), and clinical variables (body mass index, blood pressure, and glycosylated hemoglobin/HbA1c) were collected at baseline. iDAT app use trajectories were delineated using latent-class growth modeling (LCGM). Association of patient characteristics with the trajectories was ascertained using logistic regression analysis.

**Results:**

Three iDAT app use trajectories were observed: Minimal Users (66 out of 84 patients, 78.6%, with either no iDAT use at all or use only in the first 2 weeks), Intermittent-Waning Users (10 out of 84 patients, 11.9%, with occasional weekly use mainly in the first 4 weeks), and Consistent Users (8 out of 84 patients, 9.5%, with weekly use throughout all or most of the 8 weeks). The adjusted odds ratio of being a Consistent User, relative to a Minimal User, was significantly higher for females (OR 19.55, 95% CI 1.78-215.42) and for those with higher exercise motivation scores at baseline (OR 4.89, 95% CI 1.80-13.28). The adjusted odds ratio of being an Intermittent-Waning User relative to a Minimal User was also significantly higher for those with higher exercise motivation scores at baseline (OR 1.82, 95% CI 1.00-3.32).

**Conclusions:**

This study provides insight into the nature and extent of usage of a caloric-monitoring app among patients with type 2 diabetes and managed in primary care. The application of LCGM provides a useful framework for evaluating future app use in other patient populations.

## Introduction

The prevalence of type 2 diabetes mellitus is expected to rise globally with an increasingly urbanized and aging population [1]. In Singapore, prevalence among adults aged 18-69 years increased from 8.2% in 2004 to 11.3% in 2010 and is expected to continue to rise as the population gets older and more obese [2,3]. Diabetes is a chronic condition that requires patient self-management as well as continual medical care by health care providers. Patients with better self-care behaviors such as adherence to meal recommendations and glucose monitoring have been shown to develop better control of their condition than patients who were given more medications [4].

A meta-analysis of 22 trials attested to the possibility of significant reductions in glycosylated hemoglobin (Hb_A1c_) levels (mean 0.5%; 95% CI 0.3-0.7) through the self-management of diabetes using mobile phone interaction [5]. In 2012, at 74%, Singapore was the world’s leading country in smartphone penetration and by 2013, smartphone penetration had increased to 78% [6]. In terms of app usage, Singapore is presently 5^th^in the world at 75% [7]. To leverage the increasing ownership and use of smartphones and apps, the Singapore Health Promotion Board (HPB) created a mobile app called the “interactive Diet and Activity Tracker” (iDAT), which enables users to track daily calories consumed and burned using a database of locally available foods. Although the app is intended for use by anyone whether they have diabetes or not, a healthy diet, exercise, and weight loss or healthy weight maintenance are still the mainstay of first-line therapies for managing diabetes [8]. The iDAT app can function in a supportive role to aid diabetes patients in lifestyle self-management by allowing them to monitor diet and exercise.

Several studies have been conducted on the use of technology and mobile phones in diabetes management, including studies using interventional approaches—as opposed to control—whereby intervention groups received mobile phone reminders or feedback on self-monitoring of glucose levels [9,10]. However, research attempting to understand usage patterns of mobile phone-based interventions has been challenging. A few studies have attempted to assess usage patterns, but in a simplistic manner that provided minimal useful information—descriptions, averages, or tabulation of usage data [9,11]. Comparing predictors and clinical outcomes among diverse usage patterns becomes problematic owing to the difficulty in defining and distinguishing meaningful usage patterns over time.

“Latent-class growth modeling” (LCGM) is a statistical technique that exploits the existence of latent groups of individuals who share similar time trajectories of a particular trait, the characterization of which allows better understanding of the pattern of change in that variable [12,13]. LCGM has been used for some time in criminological and behavioral research, and only more recently in medicine and public health research studies of body mass trajectories in children and adults [14,15]. To our knowledge, LCGM has not been used to analyze app usage patterns in a patient population. In applying this data analysis technique, we aimed to better understand and characterize the nature and extent of technological engagement with a caloric-monitoring mobile health app (iDAT) by type 2 diabetes patients in Singapore’s primary care setting—an app that could be helpful for self-management of their chronic condition.

The primary aim of our study was to assess iDAT app usage in patients with type 2 diabetes. More specifically, our goal was to identify and characterize short-term (8-week) trajectories of use of the iDAT app among patients with type 2 diabetes mellitus in a primary care setting in Singapore and to identify patient characteristics associated with different trajectories.

## Methods

### Study Design, Site, and Population

The study was conducted at one of the 18 public primary care clinics (polyclinics) located in the northeastern part of Singapore. It is a typical polyclinic, which managed almost 5000 patients with type 2 diabetes in 2013. Patients enrolled for the study had to meet all of the following inclusion criteria: (1) above 21 years of age, (2) type 2 diabetes mellitus diagnosed based on World Health Organization criteria [16], (3) ownership of smartphone that is able to download the iDAT app (ie, Apple iOS or Android platform only), and (4) able to understand and use the iDAT app. Exclusion criteria were patients with (1) significant physical and/or cognitive impairment, (2) type 1 diabetes mellitus, (3) pregnant, or (4) prior use of the iDAT app.

**Figure 1:**
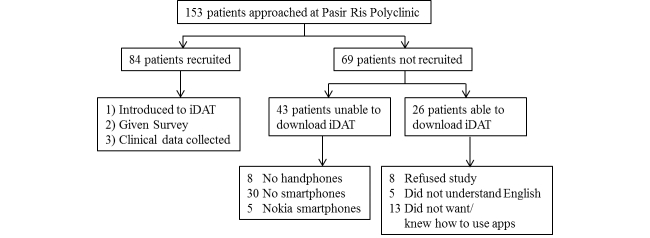
Recruitment and study flowchart.

**Figure 2:**
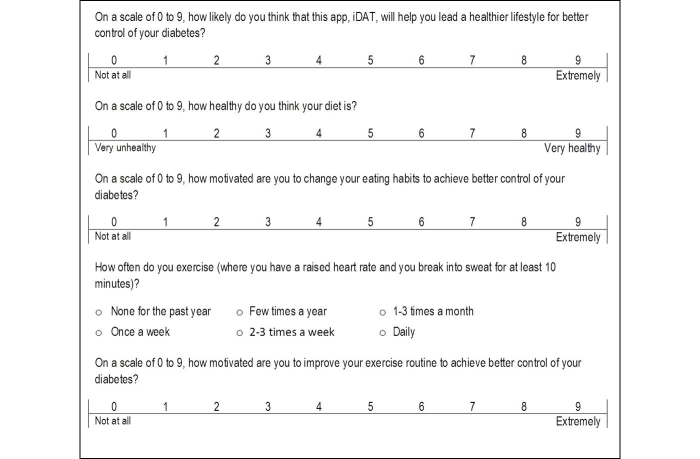
Questions evaluating "interactive Diet and Activity Tracker" (iDAT) app usefulness, current diet and exercise, motivation to improve diet, and motivation to exercise.

**Figure 3:**
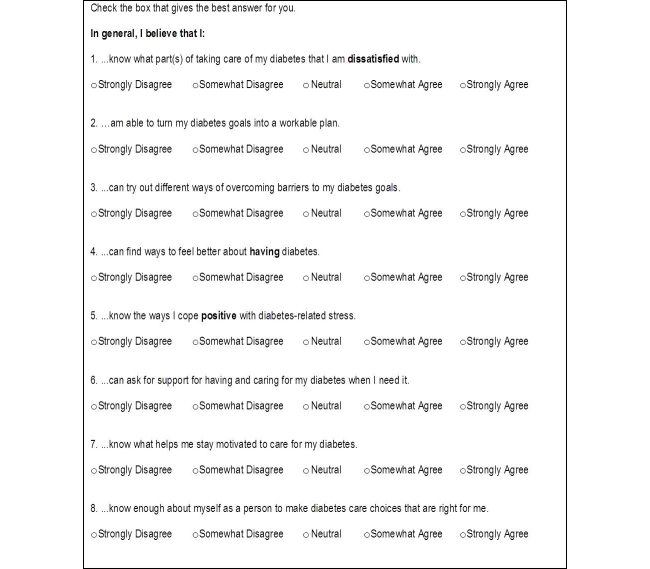
Diabetes Empowerment Scale-Short Form (DES-SF) questions developed and validated by the Michigan Diabetes Research and Training Center.

Participants were enrolled over a 5-month period from November 2013 to March 2014. Patients attending the diabetes counselling and screening services for eye and foot complications at the polyclinic were approached. Patients who declined participation, did not feel comfortable using apps, or could not understand English were not recruited (Figure 1). Recruited participants were introduced to the iDAT app and taught how to use it to monitor food intake and physical activity. Personal email addresses were used for app registration, and monitoring of app usage was based on the email address provided. A questionnaire was administered that included demographic questions, scale-based questions evaluating iDAT app usefulness, current diet and exercise, motivation to improve diet, and motivation to exercise (Figure 2). The questionnaire also included an 8-question instrument, the Diabetes Empowerment Scale-Short Form (DES-SF), developed and validated in a group of 239 African American subjects by the Michigan Diabetes Research and Training Center (Figure 3) [17]. This instrument is graded on a score of 1 (low self-efficacy) to 5 (high self-efficacy) and allows for an assessment of patients’ diabetes-related self-efficacy [17]. Patients’ clinical data including height, weight, blood pressure, and Hb_A1c_were also collected. The questionnaire was primarily self-administered, with assistance from the researcher as needed. Initially, the plan was to recruit only newly diagnosed patients. But due to slow recruitment and to reach our preliminary target of 80 patients, recruitment was expanded, 3 months into the study, to all patients who otherwise satisfied the inclusion criteria.

Patient use of the iDAT app was monitored weekly over a period of 2 months post-enrollment. There were no financial reimbursements to the patients for study participation. This study was approved by the SingHealth Centralized Institutional Review Board E (CIRB) (Ref: 2013/743/E), in accordance with all applicable regulations, and informed consent was obtained after the nature and possible consequences of the study were explained. Participants were informed when consent was taken and in the Participant Information Sheet that the email addresses used for iDAT registration would be collected and used to track app usage. This personal information, together with the other data collected, were to be kept confidential and only used on a need-to-know basis as approved by the CIRB.

### iDAT Smartphone App

**Figure 4:**
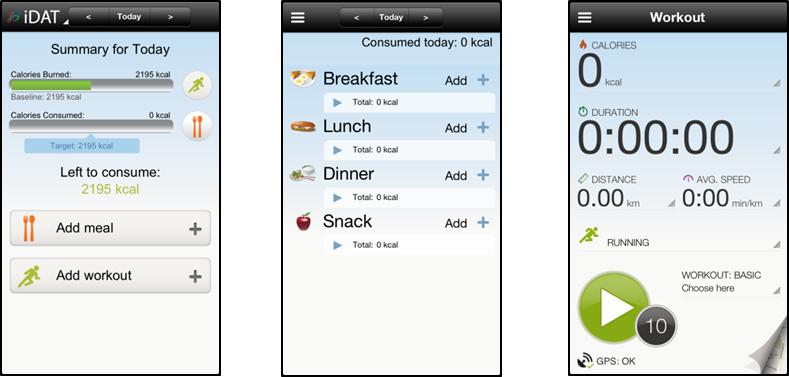
"interactive Diet and Activity Tracker" (iDAT) screen captures.

The iDAT app (Figure 4) was developed by the HPB, a statutory board under the Ministry of Health, Singapore, established to drive national health promotion and disease prevention programs. The iDAT app, though not diabetes-specific, was chosen because it was created for the local Singapore population, is freely and easily available on the two most common smartphone platforms (iOS and Android), targets first-line diabetes management of diet and exercise, and does not require any additional devices like glucometers for patients to fully utilize the app.

The app is free to download through Apple’s iTunes/App Store and Android’s Google Play. It functions as a calorie counter, helping users to balance calories consumed with calories burned on a daily basis. The “Meal” section allows the user to input food consumed via a food database with their estimated calories, including local ethnic foods. The “Workout” section enables the user to tap on their smartphones’ Global Positioning System (GPS) to monitor fitness workouts and calculate estimated calories burned. Workouts can be added manually or by using the app’s “Step Counter”. Other functions include social features such as Facebook-sharing and a “Weight and Goal” feature that allows users to set a weight loss goal and track weight loss over time.

### Statistical Analysis

Demographic variables and clinical characteristics at baseline were summarized as mean with standard deviation for continuous variables and counts and percentages for categorical variables. HPB provided the iDAT app backend information in the form of weekly use. To summarize this data, any record in terms of food entry or exercise workout entry in a week was considered as usage for that week.

A statistical analysis software (SAS) macro, PROC TRAJ, was used to apply LCGM to analyze weekly iDAT app usage data and to identify the latent groups characterizing the iDAT app use trajectories for the cohort. LCGM uses maximum likelihood to estimate model parameters [18,19]. To determine number of latent trajectory groups and trajectory shapes, multiple factors were considered: model fit statistics (Bayesian Information Criterion/BIC), significance of polynomial terms, and value of average posterior probability with an eye to parsimony in the number of trajectory groups [13,20]. The best-fit-model was chosen based on the magnitude of difference in BIC and the linear/polynomial specification that best defined the trajectory in each group, given the number of groups [18,20]. Each patient was assigned to the trajectory group for which he/she had the highest posterior probability of membership [20]. Subsequently, univariate and multivariate (stepwise) polytomous logistic regression were used to identify demographic features or clinical characteristics predictive of trajectory group membership.

## Results

### Baseline Characteristics of Recruited Patients

Of 153 patients approached, 84 who consented and satisfied the inclusion/exclusion criteria were enrolled (Figure 1). Demographics, clinical and diabetes-related variables, social lifestyle factors, smartphone characteristics, scores for motivation, and DES-SF at baseline are presented in Table 1. The mean age of the study participants was 48.2 (SD 8.5) years, with a nearly equal gender distribution. In terms of ethnic composition, 54% (45/84) were Chinese, 27% (23/84) Malays, 12% (10/84) Indians, and Other ethnicities made up the remaining 7% (6/84). Most were married (83%, 70/84) and employed (83%, 70/84). The largest group (46%, 39/84) had educational qualification of secondary school or below, followed by diploma (21%, 18/84), degree (20%, 17/84), and post-secondary school education (12%, 10/84). Their mean Body Mass Index (BMI) was 29.1 (SD 6.1) kg/m^2^, mean systolic blood pressure was 130.5 (SD 18.5) mmHg, mean diastolic blood pressure was 77.6 (SD 10.9) mmHg, and mean Hb_A1c_level was 8.7 (SD 2.5) %. A minority smoked (15%, 13/84) while 31% (26/84) were regular or social drinkers.

As we prioritized the enrollment of newly diagnosed patients, only 21% (18/84) of the enrolled participants had been diagnosed with diabetes more than a year prior to enrollment. Therefore, most of the participants had “diet only” treatment without medications (31%, 26/84) or were using one diabetes medication but not insulin (42%, 35/84).

When asked to rate how healthy their diet was on a scale of 0-9 (0-very unhealthy and 9-very healthy), the participants reported a mean score of 4.8 (SD 1.9). Their reported exercise frequency ranged from 25% (21/84) who stated they have “not exercised for the past year” to 7% (6/84) who indicated that they exercise “between 1 to 3 times per month”. They were generally quite motivated to improve their diet and exercise, giving similar mean scores of 7.3 (SD 1.5) and 6.7 (SD 1.5) respectively when asked to rate their motivation on a 0-9 scale.

Most owned Android-based smartphones (70%, 59/84). Most used their smartphones and apps frequently, with 87% (73/84) indicating that they used their smartphones more than 5 times a day and 76% (64/84) used apps more than 5 times a day. After being shown how to use iDAT, they gave a positive baseline rating for its usefulness with mean score of 6.7 (SD 1.5) on a 0-9 scale.

**Table 1:** Characteristics of enrolled patients at baseline.

Characteristic			Total recruited (n=84)
**Demographics**			
	Age (years), mean (SD)		48.2 (8.5)
	**Gender, n (%)**		
		Male	43 (51)
		Female	41 (49)
	**Ethnicity, n (%)**		
		Chinese	45 (54)
		Malay	23 (27)
		Indian	10 (12)
		Others	6 (7)
	**Marital status, n (%)**		
		Single	10 (12)
		Married	70 (83)
		Divorced / Separated	4 (5)
	**Occupational status, n (%)**		
		Retired	6 (7)
		Homemaker	7 (8)
		Unemployed	1 (1)
		Employed	70 (83)
	**Educational level, n (%)**		
		Secondary and below	39 (46)
		Post-secondary (‘A’ levels, technical)	10 (12)
		Diploma	18 (21)
		Degree and above	17 (20)
**Clinical variables, mean (SD)**			
	BMI (kg/m^2^)		29.1 (6.1)
	Height (cm)		163.7 (8.7)
	Weight (kg)		78.3 (18.9)
	**Blood pressure (mmHg), mean (SD)**		
		Systolic	130.5 (18.5)
		Diastolic	77.6 (10.9)
**Diabetes-related variables, n (%)**			
	**Time of diagnosis**		
		New (less than 1 year)	66 (79)
		Long-term (more than 1 year)	18 (21)
	**Diabetes treatment**		
		Diet only	26 (31)
		On 1 diabetes medicine (without insulin)	35 (42)
		On 2 diabetes medicines (without insulin)	16 (19)
		On insulin	7 (8)
**Social lifestyle**			
	Healthy diet score (0-9), mean (SD)		4.8 (1.9)
	**Smoking status, n (%)**		
		No	63 (75)
		Ex-smoker	8 (10)
		Yes	13 (15)
	**Drinking status, n (%)**		
		Non-drinkers	53 (63)
		Used to drink	5 (6)
		Regular/social drinkers	26 (31)
	**Exercise frequency, n (%)**		
		None in the past year	21 (25)
		Few times per year	13 (15)
		1-3 times per month	6 (7)
		Once per week	18 (21)
		2-3 times per week	18 (21)
		Daily	8 (10)
**Smartphone characteristics, n (%)**			
	**Smartphone operating system**		
		Apple	25 (30)
		Android	59 (70)
	**Smartphone usage**		
		More than 5 times /day	73 (87)
		Less than 5 times /day	11 (13)
	**Apps usage**		
		More than 5 times /day	64 (76)
		Less than 5 times /day	20 (24)
		iDAT^a^usefulness score (0-9), mean (SD)	6.7 (1.5)
**Motivation and self-efficacy scales, mean (SD)**			
	Diet motivation score (0-9)		7.3 (1.5)
	Exercise motivation score (0-9)		6.7 (1.5)
	DES-SF^b^(1-5)		4.1 (0.5)

^a^iDAT: interactive Diet and Activity Tracker

^b^DES-SF: Diabetes Empowerment Scale-Short Form

### Weekly iDAT App Use Latent Trajectory Groups

**Figure 5:**
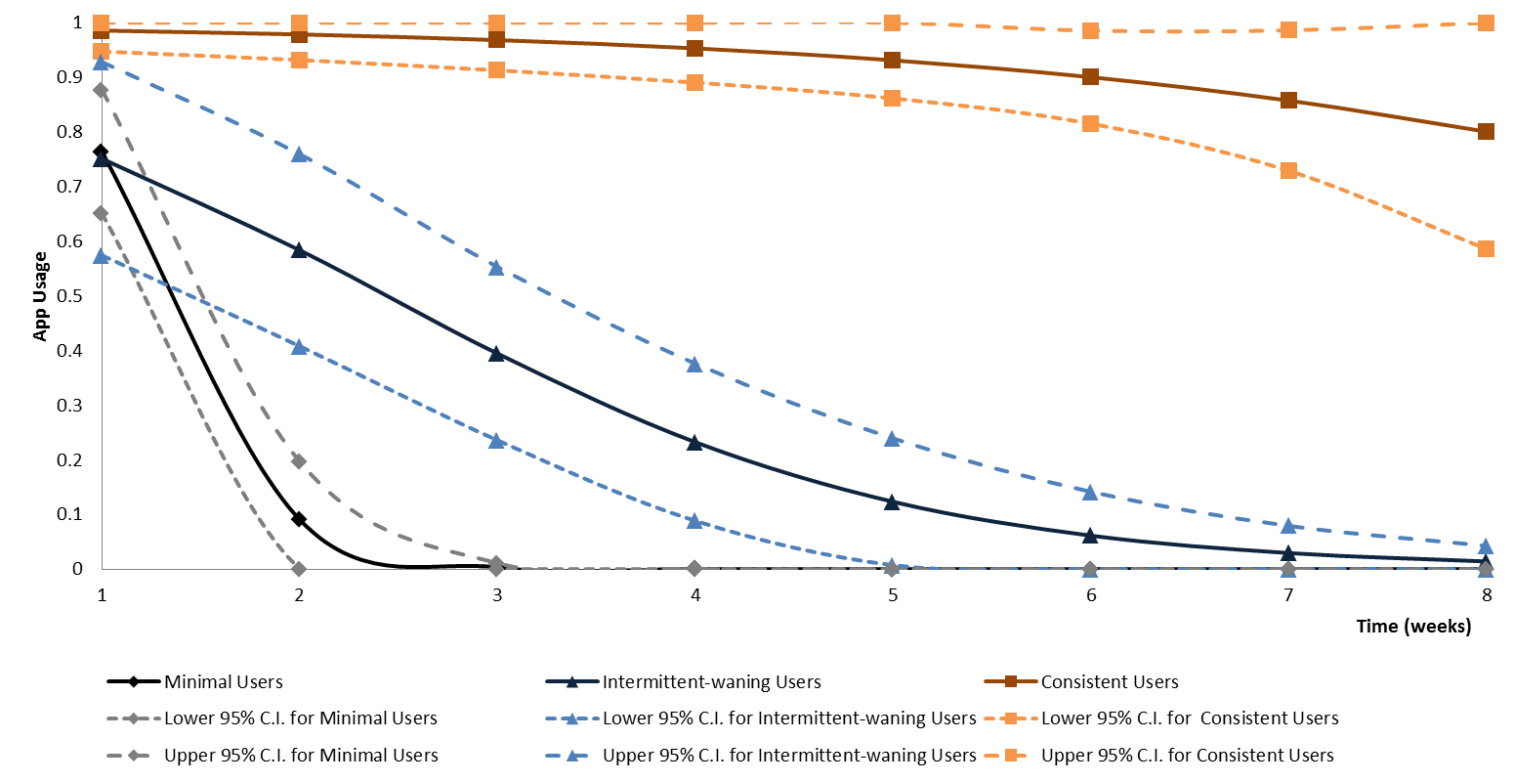
Weekly "interactive Diet and Activity Tracker" (iDAT) app use trajectory groups identified using latent class growth modeling.

Using the LCGM approach and applying goodness-of-fit criteria, weekly iDAT app use was best characterized as three latent trajectory groups (Figure 5). The model specifying four trajectory groups failed to converge and the model postulating two underlying trajectory groups had a higher BIC value indicating a poorer fit. Based upon the shape of the iDAT app use trajectory for each group, the three latent trajectory groups were labelled “Minimal Users”, “Intermittent-Waning Users”, and “Consistent Users”.

A total of 78.6% (66/84) of study participants were Minimal Users with a typical usage pattern of no iDAT input or iDAT input only during the first 2 weeks post-recruitment; 11.9% (10/84) were Intermittent-Waning Users with a typical input pattern of an occasional weekly input, mainly in the first 4 weeks post-recruitment. The remaining 9.5% (8/84) were Consistent Users with a typical input pattern of weekly input throughout all or most of the 8-week post-recruitment period.

### Predictors of Trajectory Group Membership

Univariate (Table 2) and multivariate (Table 3) polytomous logistic regression (Minimal Users as the reference category) was used to assess the baseline characteristics deemed most plausible as predictors of trajectory group membership. Univariate analysis showed that healthy diet (OR 1.6, 95% CI 1.0-2.5) and exercise motivation (OR 3.9, 95% CI 1.6-9.6) were associated with Consistent Users, and that exercise motivation (OR 1.8, 95% CI 1.0-3.1) and DES-SF scores (OR 6.6, 95% CI 1.4-29.8) were associated with Intermittent-Waning Users. The multivariate analysis resulted in two significant predictors: females had higher odds of being Consistent Users (OR 19.55, 95% CI 1.78-215.42) than males, and subjects with higher exercise motivation scores at baseline had higher odds of being Intermittent-Waning Users (OR 1.82, 95% CI 1.00-3.32) and Consistent Users (OR 4.89, 95% CI 1.80-13.28).

**Table 2:** Univariate polytomous logistic regression for baseline predictors of iDAT app use trajectory group membership (odds ratios are calculated with Minimal Users as the reference group).

		Minimal Users (n=66)	Intermittent-Waning Users (n=10)			Consistent Users (n=8)			Overall *P* value
		n (%) or mean (SD)	n (%) or mean (SD)	OR (95% CI)	*P*	n (%) or mean (SD)	OR (95% CI)	*P*	
Gender (female)		30 (45%)	4 (40%)	0.8 (0.2-3.1)	.75	7 (88%)	8.4 (1.0-72.1)	.052	.14
Age, years		47.8 (8.7)	47.2 (7.7)	1.0 (0.9-1.1)	.82	52.0 (7.7)	1.1 (1.0-1.2)	.20	.41
Body Mass Index (BMI)		29.1 (6.3)	31.0 (5.7)	1.0 (0.9-1.2)	.38	26.3 (4.2)	0.9 (0.8-1.1)	.20	.27
Glycosylated hemoglobin (Hb_A1c)_		8.9 (2.5)^a^	7.2 (1.6)	0.7 (0.4-1.0)	.07	8.5 (3.0)^b^	0.9 (0.7-1.3)	.71	.19
iDAT^c^usefulness score (0-9)		6.6 (1.6)	7.2 (1.1)	1.4 (0.8-2.3)	.21	7.4 (1.2)	1.5 (0.9-2.7)	.15	.20
Healthy diet score (0-9)		4.6 (1.9)	5.2 (1.5)	1.2 (0.8-1.7)	.39	6.1 (2.1)	1.6 (1.0-2.5)	.045	.11
Diet motivation score (0-9)		7.1 (1.6)	7.7 (0.9)	1.4 (0.8-2.3)	.25	8.3 (1.0)	2.1 (1.0-4.5)	.055	.10
**Exercise frequency**									.32
	Few times or none per year	29 (44%)	4 (40%)	0.6 (0.1-2.7)	.49	1 (13%)	0.1 (0.1-1.1)	.06	
	1-4 times per month	20 (30%)	2 (20%)	0.4 (0.1-2.6)	.36	2 (25%)	0.3 (0.1-2.0)	.23	
	More than once a week	17 (26%)	4 (40%)	Ref		5 (62%)	Ref		
Exercise motivation score (0-9)		6.4 (1.5)	7.4 (1.3)	1.8 (1.0-3.1)	.049	8.3 (0.9)	3.9 (1.6-9.6)	.003	.004
DES-SF^d^(1-5)		4.0 (0.5)	4.4 (0.3)	6.6 (1.4-29.8)	.02	4.3 (0.5)	4.0 (0.8-19.4)	.09	.02

^a^n=56, not all patients had Hb_A1c_levels at baseline. ^b^n=7, not all patients had Hb_A1c_levels at baseline.

^c^iDAT: interactive Diet and Activity Tracker

^d^DES-SF: Diabetes Empowerment Scale-Short Form

**Table 3:** Multivariate polytomous logistic stepwise regression^a^for baseline predictors of iDAT^b^app use trajectory group membership with Minimal Users group as reference category.

	Intermittent-Waning Users (n=10)		Consistent Users (n=8)		Overall *P* value
	OR (95% CI)	*P*	OR (95% CI)	*P*	
Gender (female)	1.21 (0.28-5.20)	.80	19.55 (1.78-215.42)	.02	.052
Exercise Motivation Score (0-9)	1.82 (1.00-3.32)	.05	4.89 (1.80-13.28)	.002	.003

^a^SLE (Significance Level to Enter)=SLR (Significance Level to Remove)=0.20.

^b^iDAT: interactive Diet and Activity Tracker

## Discussion

### Principal Results

To our knowledge, this is the first study to apply LCGM to delineate trajectories of app usage. We were able to distinguish usage patterns of a caloric-monitoring mobile health app into three latent trajectory groups: Minimal (76.8%), Intermittent-Waning (11.9%), and Consistent Users (9.5%). While a majority of patients did not use or rarely used the app, about 20% used the app, with close to 10% using the app on a regular basis during the 8-week post-enrollment period. The adjusted odds of being a Consistent User, as opposed to a Minimal User, were significantly higher for females and for subjects with higher exercise motivation scores at baseline. The adjusted odds of being an Intermittent-Waning User were also significantly higher for those with higher exercise motivation scores at baseline. The application of LCGM allowed us to delineate distinct trajectories of iDAT app usage and then identify predictors of specific patterns of app use.

### Comparison With Prior Work

There is strong evidence that good self-management in the chronic care of diabetes leads to better outcomes of the condition [4,21,22]. To further support patients in their effort toward better diabetes control, recent studies have tried to investigate the usage and effectiveness of incorporating technologies like the Internet and mobile phones to facilitate and support self-efficacy of diabetes patients [9,11,23]. While results have generally been positive, usage rates of the technological systems vary substantially with some as low as 13% and others as high as 92% [9,23]. To some extent, this is likely due to different study designs, conditions, and device types, which leads to difficulty in comparing results and making generalizations. Furthermore, some earlier interventions required additional resources such as dedicated health care personnel to operate a personalized messaging system or gadgets for the subjects’ use that may not have been readily available outside the study. Generalizing usage patterns in a broad context is difficult as usage varies widely among individuals and over time and, in this regard, statistical methodologies such as LCGM are of great value in identifying, segregating, and characterizing underlying latent behavioral trajectories.

Our study protocol initially aimed at enrolling newly diagnosed diabetes patients, defined as patients in their first year following a diagnosis of diabetes. We felt these patients would benefit most from using the iDAT app, since they would likely be learning new diets and making lifestyle changes. In addition, there have been few studies focused on patient self-motivation in newly diagnosed diabetes [24]. As the study progressed, we found ourselves unable to recruit sufficient numbers of patients in their first year following diagnosis and so we expanded the inclusion criteria to include patients diagnosed with diabetes longer than one year prior to enrollment. Hence, our study cohort includes a disproportionate number of newly diagnosed diabetes patients. Most participants were on no diabetes medication or only a single medication, and the average participant age was younger than that of the overall population of individuals with diabetes in Singapore [25]. However, we found no association between duration of disease and iDAT app trajectory group membership.

The younger relative age of our study cohort could also be attributed in part to the larger proportion of the younger generation owning mobile phones or being familiar with app usage. The larger representation of Indians and Malays among our study participants was consistent with the demographic profile of patients with diabetes in Singapore’s multi-ethnic population [25].

In appraising patient clinical characteristics, it was not surprising that average BMI in our study was in the “high risk category”. Obesity is a well-known risk factor for diabetes and urbanized Singapore has a rising obesity trend [2]. Average Hb_A1c_level was relatively high at 8.7% (SD 2.5), indicating that some of the patients had not been meeting their Hb_A1c_targets or due to late diagnosis of diabetes.

While the medical literature has not been clear in reflecting the differences in app usage between genders, this topic has been thoroughly analyzed in marketing research studies so that app development could be directed toward a targeted audience. Their results have shown that, while well-known and popular apps like Facebook and Twitter have equal gender usage, there are differences in the type of apps that males and females download or use [26-29]. For example, Flurry Analytics, a provider of mobile business data, found that males were more likely to download sports and automotive-related content while females downloaded more shopping catalogue apps [26]. AppAware, an app company that specializes in recommending apps for Android users, published an infographic in 2012 showing that males used more system tools while females preferred word, brain, and bubble shooting games [27]. However, in the category of health and fitness apps, results are not so definitive. According to data from Apsalar, a mobile analytics and advertising company, males used health and fitness apps 10% more than females, but information from Flurry Analytics showed that women were more likely to download health and fitness apps [26,28]. Nevertheless, this demonstrates that gender can play a role in determining app usage and a caloric-monitoring app like iDAT may appeal more to females. Further in-depth, gender-specific research may be required to determine why females have a greater preference towards iDAT or a particular type of app.

Our findings also showed that patients with higher exercise motivation scores had greater app usage. There are many barriers to initiating or increasing an exercise routine, so patients who indicate higher exercise motivation may be more determined to take active steps toward improving their diabetes, including more diligent use of the app.

### Strengths and Limitations

Implementation of this study in a primary care environment void of external pressure or add-on facilitation such as regular reminders, reimbursements, or financial incentives for participants to use the iDAT app, underpins its strength. The study provides insight into the potential of a typical mobile phone app to reach out to a target group of users in a patient population. We believe our results provide a good indication of the extent and pattern of use of this caloric-measuring app based largely on self-motivation, in a naturalistic “real-world” setting.

The analysis was limited by the fact that the app database could only provide information on app usage on a weekly basis. This limitation was considered during the study design process and was accepted on the basis of what we felt were realistic expectations for participant compliance and diligence in entering data. A database with daily app input would likely have enabled a more detailed picture of usage patterns, assuming adequate participant compliance for daily data entry.

The study has practical implications and applications. Health care providers who recommend health-related apps alongside diet and exercise instructions should be aware that only 2 in 10 are likely to use the apps and only 1 in 10 is likely to be a consistent user. Males and those with lower motivation for exercise are less likely to be frequent users of such apps. Further research is needed to understand the user’s psychological construct in the three trajectory groups, which will influence their app adoption. The design, features, and functionalities of the respective app are other potential factors that can facilitate or hinder the user’s engagement with the app and this requires further investigation.

This inaugural study using LCGM as the modality of analysis is limited by the relatively small study sample and short length of observation. However, the information gathered, especially the variations in uptake of the app across the three trajectory groups will inform the design and sample size estimation of a future study to determine the effectiveness of a caloric-measuring mobile phone app on clinical outcomes among users with diabetes.

### Conclusions

Our successful, novel application of the statistical method, LCGM, provides insightful analysis of longitudinal data to determine app utility among a target population. For selected patients with diabetes, the iDAT app can serve as an adjunct tool to facilitate lifestyle changes in conjunction with the usual modality of counselling.

## Abbreviations

BIC: Bayesian information criterion
BMI: body mass index
CIRB: Centralized Institutional Review Board
DES-SF: Diabetes Empowerment Scale-Short Form
GPS: Global Positioning System
HbA1c: glycosylated hemoglobin
HPB: Health Promotion Board
iDAT: interactive Diet and Activity Tracker
iOS: Apple operating system
LCGM: latent-class growth modeling
SLE: significance level to enter
SLR: significance level to remove
